# BACTERIAL PNEUMONIA IS A POSSIBLE RISK FACTOR FOR ORAL CANDIDIASIS IN OLDER ADULTS: A RETROSPECTIVE COHORT STUDY.

**DOI:** 10.1093/geroni/igz038.3189

**Published:** 2019-11-08

**Authors:** Masato Nakajima, Yojiro Umezaki, Masahiro Yamaguchi, Michiko Makino, Nao suzuki, Masahiro Yoneda, Takao Hirofuji, Hiromitsu Morita

**Affiliations:** 1 Department of General Dentistry, Fukuoka Dental College, Fukuoka, Japan; 2 Department of Preventive and Public Health Dentistry, Fukuoka Dental College, Fukuoka, Japan

## Abstract

Both aspiration pneumonia and oral candidiasis are caused by opportunistic infection of intraoral commensals and have many similar risk factors linked to oral health in older adults. Candida albicans forms biofilms with respiratory bacteria such as Klebsiella pneumoniae and Pseudomonas aeruginosa. The aim of our study was to determine the relationship between bacterial pneumonia, including aspiration pneumonia, and oral candidiasis in older patients who were hospitalized with several systemic diseases in a community-based acute care hospital without a dental unit. We retrospectively analyzed 228 older patients (male: 105, female: 123) using multiple logistic regression. The mean age of the patients was 81.3 (SD: 11.1) years. Forty-four patients were oral candidiasis positive, and 78 patients suffered from bacterial pneumonia, including aspiration pneumonia. Results showed that bacterial pneumonia had the strongest statistical relationship with oral candidiasis (p=0.000, OR: 5.173, 95 % CI: 2.368–11.298). This was followed by poor oral hygiene (p=0.001, OR: 6.095, 95 % CI: 2.003–18.545) and severe dry mouth (p=0.043, OR: 2.507, 95 % CI: 1.031–6.098). Other conventional risk factors for oral candidiasis, such as diabetes mellitus, denture wearer, dysphagia, malnutrition, requiring care, use of inhalation steroids, were not statistically significant in this study. Fifteen species of bacteria, including Klebsiella pneumoniae and Pseudomonas aeruginosa, were detected by pharyngeal sputum culture in 24 of 31 patients who were diagnosed with both oral candidiasis and bacterial pneumonia. In conclusion, bacterial pneumonia is a possible new risk factor for oral candidiasis in older adults.

